# Supplementary material for: Chromothripsis during telomere crisis is independent of NHEJ, and consistent with a replicative origin
Source: Genome Res. 2019 May;29(5):737–49. doi: 10.1101/gr.240705.118 (PMC6499312; doi:10.1101/gr.240705.118)
Supplement: Supplemental Material [file supp_gr.240705.118_Supplemental_file_1.zip › contigs/annotated_contigs/DB90/contig.2.DB90_length_301_mean_cov_4.48504983389.docx]

**DB90_length_301_mean_cov_4.48504983389**

```
AAAGAATGCTTCTTTCTTAGACTATACACAATGTGGCAAAACTGGGAAATGAAACAAGGAAACTTTCCCACATCCAAGTCACCACTAAA
 >chr3:132278805-132279017 + E=2e-116
ACAGGATCTTTAACAAAAGCCAACAAAGAGATCCTGCTGCAATGCCATATTTCAGTCAATACAGACCCTCTGGAAAGAGACACCTCTTC

CAAATCACATTTTATCCATTTATTTCTAACTT|GT|TTAGCCGGCCCAGCGAGGAAGGAAGCCGAAAGAGCCAGGTTTTTGATGAGGGG
                                 >chr3:132379101-132379192 + E=2e-43
GAGCGATGTCTGCGACGCACCGGAAGCGGCTCCGAG
```
